# Effect of coded steatotic liver disease on outcomes of percutaneous coronary intervention in non-diabetic adults: insights from a multicenter real-world cohort

**DOI:** 10.1186/s12872-026-06017-y

**Published:** 2026-05-25

**Authors:** Yussif Issaka, Mohammad Maraqah, Didien Meyahnwi, Kwame Adjei-Sefah, Efeturi Okorigba, Miracle Oparah, Sarpong Boateng, Hakeemah Abdul-Kareem, Mayowa Adefuye, Guy Loic Nguefang, Basile Njei, Samuel Hahn, Stuart Zarich

**Affiliations:** 1 Department of Internal Medicine, Bridgeport Hospital, Yale New Haven Health, Bridgeport, CT USA; 2 College of Medicine, Hebron University, Hebron, Palestine; 3 Department Of internal Medicine, West Virginia University School of Medicine, Morgantown, WV USA; 4 Section of Digestive Diseases, Department of Medicine, Yale School of Medicine, New Haven, Connecticut USA; 5 University of Ghana Medical Center, Accra, Ghana; 6 University of Alabama at Birmingham, Birmingham, AL USA; 7 Texas Tech university health Sciences Center, Odessa, TX USA; 8 Department of Cardiovascular Medicine, Bridgeport Hospital /Yale New Haven Health, Bridgeport , United States

**Keywords:** Steatotic liver disease, MASLD, Percutaneous coronary intervention, Heart failure, Bleeding, Cardiogenic shock, Cardiovascular outcomes, TriNetX

## Abstract

**Background:**

Metabolic dysfunction-associated steatotic liver disease (MASLD) is a cardiovascular risk–enhancing condition. Its prognostic significance following PCI in non-diabetic adults, and the temporal distribution of that risk, remain incompletely characterized.

**Objectives:**

To evaluate whether coded steatotic liver disease is associated with adverse one-year post-PCI outcomes in non-diabetic adults, and to characterize the temporal concentration of risk across early (day 0–30) and late (day 31–365) post-procedural periods.

**Methods:**

Retrospective cohort study using the TriNetX US Collaborative Network (2014–2023). Non-diabetic adults undergoing PCI were stratified by steatotic liver disease (SLD) status (ICD-10-CM K76.0 and/or K75.81). Primary outcome: all-cause mortality (day 1–365). Secondary outcomes: heart failure, MI, 3-point MACE, cardiogenic shock, cardiac arrest, and bleeding. PSM (1:1) balanced demographics, cardiometabolic comorbidities, ACS type, and medications. Outcomes were further assessed across prespecified early and late windows.

**Results:**

After PSM, 7,434 patients per group were well balanced (SMD < 0.10). All-cause mortality did not differ significantly (2.8% vs. 3.1%; HR, 0.93; 95% CI, 0.77–1.13; *p* = 0.472). SLD was significantly associated with higher one-year rates of incident heart failure (10.1% vs. 8.9%; HR, 1.15; 95% CI, 1.02–1.30; *p* = 0.027) and bleeding events (5.1% vs. 3.8%; HR, 1.35; 95% CI, 1.15–1.59; *p* < 0.001). Time-stratified analysis showed that cardiogenic shock risk was concentrated in the first 30 days (RR, 1.45; *p* < 0.001) and attenuated to null beyond 30 days and overall. Heart failure excess was similarly early-driven (RR, 1.17; *p* = 0.005), but the overall one-year signal remained significant (HR, 1.15; *p* = 0.027), reflecting the sustained contribution of early events.

**Conclusions:**

Among non-diabetic adults undergoing PCI, coded steatotic liver disease is associated with significantly higher one-year rates of heart failure and bleeding, without a significant mortality difference. Hemodynamic vulnerability is concentrated in the early peri-procedural period, while hemorrhagic risk is sustained. These findings support heightened early surveillance and individualized antithrombotic planning for SLD patients undergoing PCI.

**Supplementary Information:**

The online version contains supplementary material available at 10.1186/s12872-026-06017-y.

## Introduction

 Metabolic dysfunction–associated steatotic liver disease (MASLD), formerly classified as nonalcoholic fatty liver disease (NAFLD), is the most prevalent chronic liver disease worldwide and a growing contributor to cardiovascular morbidity and mortality [1–4]. Its prevalence has risen from approximately 21.9% in the 1990s to an estimated 37.3% by 2019, with projections exceeding 41.4% by 2050 [3, 5, 6]. In the United States, MASLD is projected to affect over 100 million adults within this timeframe [6].

Beyond its hepatic manifestations, MASLD is recognized as a systemic metabolic disorder with significant cardiovascular implications [7, 8]. Individuals with MASLD carry a two- to threefold higher risk of coronary artery disease and an approximately 35% greater risk of cardiovascular mortality compared to those without hepatic steatosis [1, 9–12]. Key mechanistic drivers include systemic inflammation, proatherogenic dyslipidemia, endothelial dysfunction, and direct myocardial effects mediated via the liver–heart axis [13–18]. MASLD is also associated with a fragile hemostatic state- involving impairment of procoagulant and anticoagulant pathways, platelet dysfunction, and defective fibrinolysis, that may alter thrombotic and hemorrhagic risk in procedural settings.

Percutaneous coronary intervention (PCI) is among the most performed cardiovascular procedures, and periprocedural risk stratification remains a clinical priority. While diabetes mellitus is a well-established predictor of worse PCI outcomes [19], the contribution of MASLD in the absence of overt diabetes remains incompletely characterized. Prior studies have been limited by the inclusion of diabetic patients, variable MASLD ascertainment, and a predominant focus on in-hospital endpoints [1, 20, 21] EHR-based studies are further constrained by the inability to verify the full contemporary MASLD definition from administrative coding [22]. We conducted a large, propensity score–matched retrospective cohort study using the TriNetX US Collaborative Network to evaluate the association between coded steatotic liver disease and one-year post-PCI outcomes in non-diabetic adults, with prespecified temporal stratification to distinguish acute peri-procedural vulnerability from sustained long-term risk.

## Methods

### Data source

This retrospective cohort study used data from the TriNetX Research Network, a federated electronic health record platform comprising 67 U.S. healthcare organizations, for the period January 1, 2014, to December 31, 2023. All data were de-identified and reported at the aggregate level. In accordance with federal regulations for research on de-identified datasets, the study was deemed exempt from Institutional Review Board oversight.

**Fig. 1:**
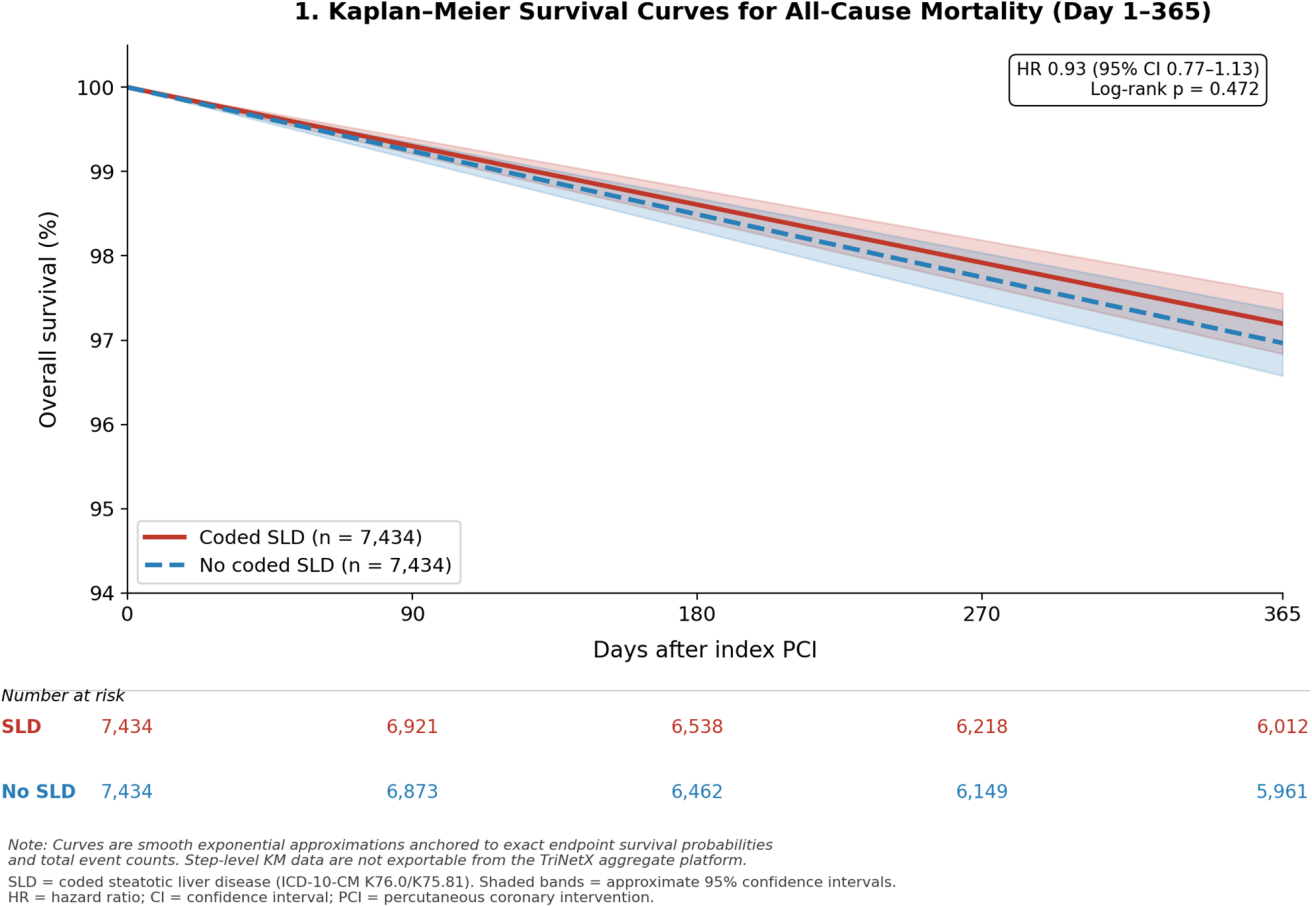
Kaplan–Meier Survival Curves for All-Cause Mortality (Day 1–365). No significant difference was observed (HR, 0.93; 95% CI, 0.77–1.13; log-rank p=0.472). SLD = steatotic liver disease. Note: Curves are smooth exponential approximations anchored to exact endpoint survival probabilities and total event counts. Step-level KM data are not exportable from the TriNetX aggregate platform. SLD = coded steatotic liver disease (ICD-10-CM K76.0/K75.81). Shaded bands = approximate 95% confidence intervals. HR = hazard ratio; CI = confidence interval; PCI = percutaneous coronary intervention

**Fig. 2:**
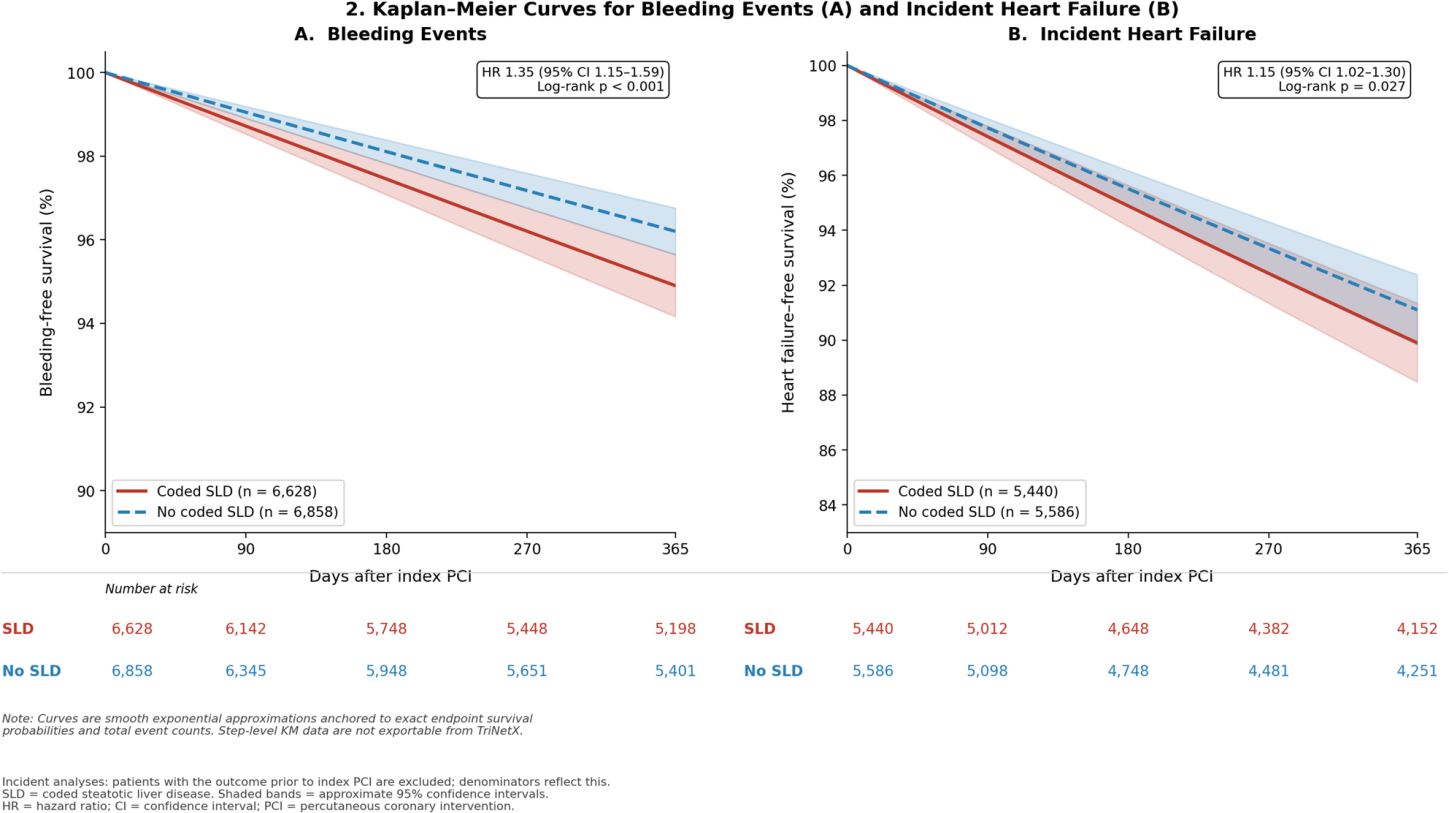
Kaplan–Meier Curves for Bleeding Events (**A**) and Incident Heart Failure (**B**) Over One Year. SLD was associated with significantly higher rates of bleeding (HR, 1.35; 95% CI, 1.15–1.59; *p* < 0.001) and heart failure (HR, 1.15; 95% CI, 1.02–1.30; *p* = 0.027). Abbreviations as in Fig. 1. Note: Curves are smooth exponential approximations anchored to exact endpoint survival probabilities and total event counts. Step-level KM data are not exportable from the TriNetX aggregate platform. Incident analyses: patients with the outcome prior to index PCI are excluded; denominators reflect this. SLD = coded steatotic liver disease. Shaded bands = approximate 95% confidence intervals. HR = hazard ratio; CI = confidence interval; PCI = percutaneous coronary intervention

**Fig. 3:**
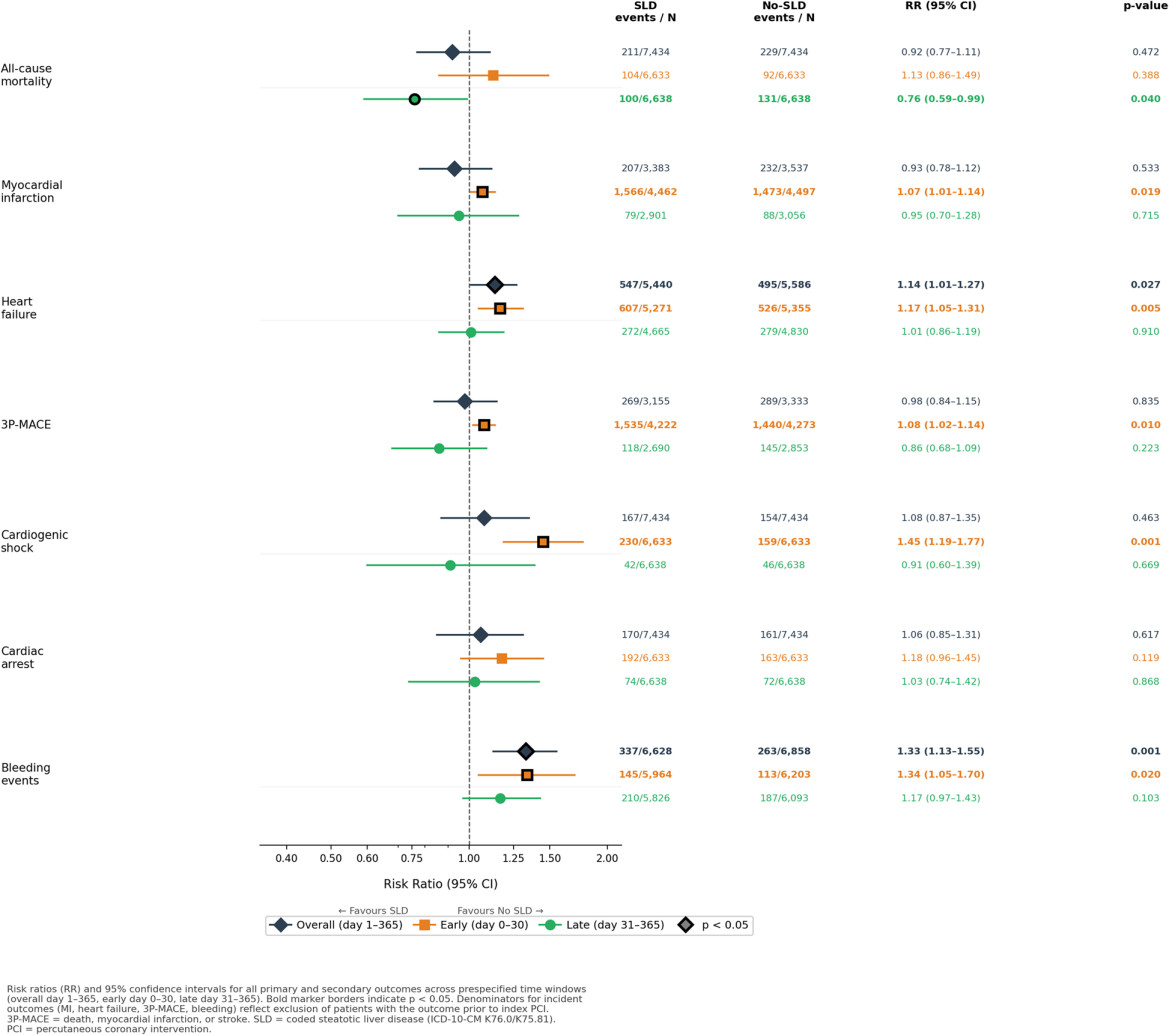
Forest Plot of Clinical Outcomes by Time Period. HRs and RRs (95% CIs) for all primary and secondary outcomes across overall (day 1–365), early (day 0–30), and late (day 31–365) windows. 3P-MACE = death, MI, or stroke. SLD = steatotic liver disease

### Study population and cohort definitions

Adults aged ≥ 18 years undergoing PCI (identified using standardized CPT codes) were eligible. Patients with diabetes mellitus (E08–E13) within five years of index PCI were excluded. The exposure cohort comprised patients with documented steatotic liver disease (K76.0 and/or K75.81 within five years prior to index PCI). This EHR-based definition captures patients across the steatotic liver disease spectrum but cannot confirm the full contemporary MASLD definition, including metabolic criteria and fibrosis staging [22, 23]. The exposure is interpreted throughout as coded steatotic liver disease rather than strictly defined MASLD. The comparator cohort comprised patients undergoing PCI without documented hepatic steatosis, steatohepatitis, or diabetes; this group likely includes individuals with unrecognized steatotic liver disease, biasing results toward the null. Both cohorts excluded patients with alcohol-related liver disease (K70.*), unspecified cirrhosis (K74.60), hepatic failure (K72), malignancy (C00–D49), or prior solid-organ transplantation (Z94, Z94.4).

### Outcomes

Primary outcome: all-cause mortality (day 1–365). Secondary outcomes: incident heart failure (I50), MI (I21, I22), 3-point MACE (death, MI, or stroke [I63]), cardiogenic shock (R57.0), cardiac arrest (I46), and bleeding events (composite of K92.0–K92.2, I60–I62, R58, I97.41, I97.410, I97.411, I97.418, I97.42, I97.610). For incident analyses (MI, heart failure, 3P-MACE, bleeding), patients with a history of the outcome prior to index PCI were excluded; denominators are reported explicitly. The bleeding composite does not permit BARC or TIMI classification, which represents a key limitation.

### Temporal stratification

Outcomes were assessed across three prespecified windows: early (day 0–30), late (day 31–365), and overall (day 1–365). Late-period analyses were restricted to patients surviving through day 30 (landmark approach).

### Propensity score matching

One-to-one PSM utilized a greedy nearest-neighbor algorithm (caliper 0.1 pooled SD). Covariates included age, sex, race, ethnicity, cardiovascular comorbidities, cardiometabolic risk factors, chronic kidney disease, liver fibrosis (K74), baseline ACS type (NSTEMI I21.4; STEMI subtypes I21.0, I21.1, I21.2, I21.3; unspecified AMI I21.9), baseline medications including individual P2Y12 inhibitors (clopidogrel, ticagrelor, prasugrel), anticoagulants, antilipemic agents, ACE inhibitors, ARBs, beta-blockers, SGLT2 inhibitors, and GLP-1 receptor agonists, and left ventricular ejection fraction (LVEF) where documented. Given that echocardiographic documentation was available in only a subset of patients, LVEF was matched on available data only. Balance was assessed using SMDs (SMD < 0.10 = adequate). Key procedural determinants (PCI indication, lesion complexity, access site, stent type, antithrombotic intensity) were unavailable, representing a major limitation. Complete procedural, exposure, outcome, and propensity score matching code definitions are provided in Supplementary Table S1.

### Statistical analysis

Binary outcomes were compared using risk differences, risk ratios (RR), and odds ratios with 95% CIs. Time-to-event analyses used Kaplan–Meier methods and Cox proportional hazards regression; HR is the primary effect measure for the overall period. The proportional hazards assumption was evaluated using Schoenfeld residuals. All tests were two-sided (significance threshold *p* < 0.05). Formal competing-risk analysis was not feasible given the aggregate-level TriNetX data structure. All analyses were performed within the TriNetX analytics platform.

## Results

### Study population and baseline characteristics

A total of 7,523 non-diabetic patients with coded steatotic liver disease and 143,843 patients without documented steatotic liver disease were identified. After 1:1 PSM, 7,434 patients remained in each cohort. Baseline characteristics were well balanced, with SMDs < 0.10 across all covariates (Table 1). The mean age was 61.9 ± 11.5 vs. 61.4 ± 12.0 years (SMD, 0.038). Approximately 67.6% of patients in both groups were male. The prevalence of baseline heart failure was 20.3% in the SLD cohort and 19.1% in the comparator cohort (SMD, 0.030). ACS type was well balanced: NSTEMI in 23.7% vs. 23.2%, STEMI (all subtypes) in approximately 10.8% vs. 11.2%. P2Y12 inhibitor use was similar across groups: clopidogrel 21.7% vs. 20.9%, ticagrelor 3.1% vs. 3.2%, prasugrel 1.9% vs. 2.0%. Liver fibrosis (K74) was present in 1.3% vs. 1.4% after matching (SMD, 0.011). LVEF was included as a PSM covariate and was documented in 1,062 SLD patients (14.3%) and 981 comparators (13.2%) after matching, with mean values of 55.8 ± 13.4% and 54.4 ± 13.6%, respectively (post-match SMD, 0.101). This marginally exceeded the prespecified < 0.10 threshold, reflecting differential echocardiographic documentation frequency across cohorts; notably, the SLD cohort had a slightly higher mean LVEF, indicating that if anything, the comparator cohort had marginally worse baseline systolic function. The mean follow-up was 328.7 days in the SLD cohort and 339.1 days in the comparator cohort.

**Table 1:** Baseline Characteristics Before and After Propensity Score Matching

Characteristic	SLD Pre-match (*n* = 7,523)	No SLD Pre-match (*n* = 143,843)	SLD Post-match (*n* = 7,434)	No SLD Post-match (*n* = 7,434)	Post-match SMD
Demographics					
Age, mean ± SD, years	61.8 ± 11.5	64.8 ± 12.3	61.9 ± 11.5	61.4 ± 12.0	0.038
Female sex, *n* (%)	2,446 (32.5%)	41,942 (29.2%)	2,412 (32.4%)	2,410 (32.4%)	0.001
White race, *n* (%)	6,288 (83.6%)	115,986 (80.6%)	6,219 (83.7%)	6,299 (84.7%)	0.030
Hispanic or Latino, *n* (%)	333 (4.4%)	4,499 (3.1%)	327 (4.4%)	310 (4.2%)	0.011
Asian, *n* (%)	230 (3.1%)	4,871 (3.4%)	228 (3.1%)	234 (3.1%)	0.005
Comorbidities					
Hypertension (I10), *n* (%)	5,696 (75.8%)	79,136 (55.0%)	5,617 (75.6%)	5,633 (75.8%)	0.005
Chronic ischemic heart disease (I25), *n* (%)	4,871 (64.8%)	76,922 (53.5%)	4,812 (64.7%)	4,757 (64.0%)	0.015
Heart failure (I50), *n* (%)	1,535 (20.4%)	23,048 (16.0%)	1,507 (20.3%)	1,419 (19.1%)	0.030
Atrial fibrillation / flutter (I48), *n* (%)	978 (13.0%)	16,984 (11.8%)	964 (13.0%)	922 (12.4%)	0.017
Cerebral infarction history (I63), *n* (%)	457 (6.1%)	5,823 (4.0%)	450 (6.1%)	416 (5.6%)	0.020
NSTEMI (I21.4), *n* (%)	1,775 (23.6%)	30,094 (20.9%)	1,761 (23.7%)	1,723 (23.2%)	0.012
STEMI — unspecified site (I21.3), *n* (%)	405 (5.4%)	7,091 (4.9%)	401 (5.4%)	414 (5.6%)	0.008
STEMI — inferior wall (I21.1), *n* (%)	204 (2.7%)	3,527 (2.5%)	203 (2.7%)	188 (2.5%)	0.013
STEMI — anterior wall (I21.0), *n* (%)	144 (1.9%)	2,868 (2.0%)	144 (1.9%)	154 (2.1%)	0.010
Obesity / overweight (E66), *n* (%)	2,867 (38.1%)	20,266 (14.1%)	2,793 (37.6%)	2,693 (36.2%)	0.028
Hyperlipidemia (E78.5), *n* (%)	4,873 (64.8%)	64,948 (45.2%)	4,801 (64.6%)	4,748 (63.9%)	0.015
Chronic kidney disease (N18), *n* (%)	887 (11.8%)	12,477 (8.7%)	873 (11.7%)	771 (10.4%)	0.039
Liver fibrosis (K74), *n* (%)	135 (1.8%)	162 (0.1%)	93 (1.3%)	102 (1.4%)	0.011
LVEF, mean ± SD (%)	55.7 ± 13.5	52.9 ± 14.8	55.8 ± 13.4	54.4 ± 13.6	0.101*
— Documented subset, *n* (%)	1,085 (14.4%)	11,263 (7.8%)	1,062 (14.3%)	981 (13.2%)	0.031
Baseline medications					
Antilipemic agents, *n* (%)	4,924 (65.5%)	71,704 (49.8%)	4,851 (65.3%)	4,834 (65.0%)	0.006
Aspirin, *n* (%)	4,551 (60.5%)	66,136 (46.0%)	4,489 (60.4%)	4,521 (60.8%)	0.008
Anticoagulants, *n* (%)	4,363 (58.0%)	61,356 (42.7%)	4,304 (57.9%)	4,198 (56.5%)	0.028
Clopidogrel, *n* (%)	1,630 (21.7%)	29,158 (20.3%)	1,613 (21.7%)	1,555 (20.9%)	0.019
Ticagrelor, *n* (%)	234 (3.1%)	5,240 (3.6%)	232 (3.1%)	238 (3.2%)	0.006
Prasugrel, *n* (%)	145 (1.9%)	2,318 (1.6%)	144 (1.9%)	150 (2.0%)	0.007
ACE inhibitors, *n* (%)	2,470 (32.9%)	34,084 (23.7%)	2,431 (32.7%)	2,419 (32.5%)	0.004
Angiotensin II inhibitors (ARBs), *n* (%)	1,858 (24.7%)	21,191 (14.7%)	1,816 (24.4%)	1,737 (23.4%)	0.024
Beta-blockers, *n* (%)	4,604 (61.2%)	52,227 (36.3%)	4,531 (61.0%)	4,455 (59.9%)	0.021
SGLT2 inhibitors, *n* (%)	273 (3.6%)	1,082 (0.8%)	256 (3.4%)	238 (3.2%)	0.013
GLP-1 receptor agonists, *n* (%)	233 (3.1%)	410 (0.3%)	184 (2.5%)	175 (2.4%)	0.006

LVEF was included as a PSM covariate; post-match SMD of 0.101 reflects differential documentation frequency (available in 14.3% SLD vs. 13.2% no-SLD after matching) rather than physiological imbalance. All other post-match SMD values < 0.10

*SLD* steatotic liver disease, *SMD* standardized mean difference, *NSTEMI* non-ST-elevation MI, *STEMI* ST-elevation MI, *CKD* chronic kidney disease, *ACE* angiotensin-converting enzyme, *ARB* angiotensin receptor blocker

### Primary outcome: all-cause mortality

Over the full one-year follow-up (day 1–365), all-cause mortality occurred in 211 patients (2.8%) in the SLD cohort and 229 patients (3.1%) in the comparator cohort (RR, 0.92; 95% CI, 0.77–1.11; HR, 0.93; 95% CI, 0.77–1.13; log-rank *p* = 0.472) Fig. 1. The difference was not statistically significant.

### Secondary outcomes: overall one-year period (Day 1–365)

Coded steatotic liver disease was significantly associated with higher one-year rates of two secondary outcomes. Incident heart failure occurred in 547 of 5,440 eligible patients (10.1%) in the SLD cohort versus 495 of 5,586 patients (8.9%) in the comparator cohort (RR, 1.14; 95% CI, 1.01–1.27; HR, 1.15; 95% CI, 1.02–1.30; *p* = 0.027). Bleeding events were significantly more frequent in the SLD cohort: 337 of 6,628 eligible patients (5.1%) versus 263 of 6,858 patients (3.8%) (RR, 1.33; 95% CI, 1.13–1.55; HR, 1.35; 95% CI, 1.15–1.59; *p* < 0.001) Fig. 2. All remaining outcomes did not differ significantly over the full year: MI (6.1% vs. 6.6%; HR, 0.94; *p* = 0.533), 3P-MACE (8.5% vs. 8.7%; HR, 1.00; *p* = 0.957), cardiogenic shock (2.2% vs. 2.1%; HR, 1.09; *p* = 0.463), and cardiac arrest (2.3% vs. 2.2%; HR, 1.06; *p* = 0.617). The results are presented in Table 2.

**Table 2:** One-Year Clinical Outcomes After PCI — Primary Matched Cohort (*n* = 7,434/group, Day 1–365)

Outcome	SLD + PCI *n*/*N* (risk)	No SLD + PCI *n*/*N* (risk)	Risk Ratio (95% CI)	Hazard Ratio (95% CI)	*p*-value
All-cause mortality†	211/7,434 (2.8%)	229/7,434 (3.1%)	0.92 (0.77–1.11)	0.93 (0.77–1.13)	0.472
Myocardial infarction‡	207/3,383 (6.1%)	232/3,537 (6.6%)	0.93 (0.78–1.12)	0.94 (0.78–1.14)	0.533
Heart failure‡	547/5,440 (10.1%)	495/5,586 (8.9%)	1.14 (1.01–1.27)	1.15 (1.02–1.30)	0.027*
3-Point MACE‡	269/3,155 (8.5%)	289/3,333 (8.7%)	0.98 (0.84–1.15)	1.00 (0.84–1.18)	0.957
Cardiogenic shock†	167/7,434 (2.2%)	154/7,434 (2.1%)	1.08 (0.87–1.35)	1.09 (0.88–1.36)	0.463
Cardiac arrest†	170/7,434 (2.3%)	161/7,434 (2.2%)	1.06 (0.85–1.31)	1.06 (0.86–1.32)	0.617
Bleeding events‡	337/6,628 (5.1%)	263/6,858 (3.8%)	1.33 (1.13–1.55)	1.35 (1.15–1.59)	< 0.001*

† All patients included regardless of prior outcome status

‡ Incident analysis: patients with the outcome prior to index PCI excluded; denominators reflect this

3P-MACE = death, MI, or stroke

* *p* < 0.05. SLD = steatotic liver disease

### Temporal analysis: early (Day 0–30) vs. late (Day 31–365)

Time-stratified analyses revealed a distinct temporal pattern (Table 3). Within the first 30 days, SLD was associated with significantly higher rates of cardiogenic shock (3.5% vs. 2.4%; RR, 1.45; 95% CI, 1.19–1.77; *p* < 0.001), heart failure (11.5% vs. 9.8%; RR, 1.17; 95% CI, 1.05–1.31; *p* = 0.005), and bleeding (2.4% vs. 1.8%; RR, 1.34; 95% CI, 1.05–1.70; *p* = 0.020), as well as modest increases in MI (RR, 1.07; *p* = 0.019) and 3P-MACE (RR, 1.08; *p* = 0.010). Beyond 30 days, no statistically significant differences were observed in cardiogenic shock, MI, 3P-MACE, or cardiac arrest. Heart failure did not reach significance in the late window alone (RR, 1.01; *p* = 0.910); however, the overall one-year heart failure signal remained significant (HR, 1.15; *p* = 0.027), driven predominantly by early-period events Table 3 and Fig. 3.

**Table 3:** Temporal Analysis — Early (Day 0–30) vs. Late (Day 31–365) vs. Overall (Day 1–365)

Outcome	Early SLD risk	Early RR (95% CI); *p*	Late SLD risk	Late RR (95% CI); *p*	Overall RR (95% CI); *p*	Temporal pattern
Cardiogenic shock	3.5%	1.45 (1.19–1.77); <0.001	0.6%	0.91 (0.60–1.39); 0.669	1.08 (0.87–1.35); 0.463	Concentrated early
Heart failure	11.5%	1.17 (1.05–1.31); 0.005	5.8%	1.01 (0.86–1.19); 0.910	1.14 (1.01–1.27); 0.027	Early-driven; overall significant
Bleeding events	2.4%	1.34 (1.05–1.70); 0.020	3.6%	1.17 (0.97–1.43); 0.103	1.33 (1.13–1.55); <0.001	Sustained; both periods
Myocardial infarction	35.1%§	1.07 (1.01–1.14); 0.019	2.7%	0.95 (0.70–1.28); 0.715	0.93 (0.78–1.12); 0.533	Early peri-procedural only; overall null
3-Point MACE	36.4%§	1.08 (1.02–1.14); 0.010	4.4%	0.86 (0.68–1.09); 0.223	0.98 (0.84–1.15); 0.957	Early peri-procedural only; overall null
All-cause mortality	1.6%	1.13 (0.86–1.49); 0.414	1.5%	0.76 (0.59–0.99); 0.040‡	0.92 (0.77–1.11); 0.472	Null overall; survival effect late

*SLD* steatotic liver disease, *RR* risk ratio

§ High early MI / MACE rates reflect index hospitalization coding and peri-procedural period, not independent new events

‡ Late-period mortality RR 0.76 most plausibly reflects a survivor effect. For the late period, analyses were restricted to patients surviving through day 30

## Discussion

In this large, propensity score–matched cohort of non-diabetic adults undergoing PCI, coded steatotic liver disease was associated with significantly higher one-year rates of incident heart failure and bleeding events, without a significant difference in all-cause mortality over the full year. Time-stratified analyses revealed that hemodynamic vulnerability, particularly cardiogenic shock and heart failure, was concentrated in the early peri-procedural period (day 0–30), with no significant excess beyond 30 days. Bleeding risk showed a more sustained pattern across both periods. These findings characterize a clinically meaningful but temporally nuanced risk profile: early hemodynamic fragility and persistent hemorrhagic vulnerability.

### Heart failure and the liver–heart axis

The significant association between coded SLD and one-year incident heart failure (HR, 1.15; 95% CI, 1.02–1.30) is the most robust finding of this analysis Fig. 2B. MASLD has been associated with myocardial steatosis and impaired energetics, systemic cytokine activation, coronary microvascular dysfunction, and increased ventricular stiffness [14, 15]. These processes may reduce cardiovascular reserve and increase susceptibility to hemodynamic decompensation following PCI, particularly during ischemia-reperfusion injury. Multiple investigations have established that noninvasive markers of liver fibrosis independently predict heart failure risk, with MASLD conferring approximately 1.5-fold elevated long-term risk [13, 24]. Biccirè et al. [25] demonstrated that FIB-4–detected liver fibrosis was independently associated with in-hospital adverse events, including cardiogenic shock and heart failure concentrated during the acute hospitalization following PCI. The temporal concentration of the heart failure signal in the first 30 days in our study is consistent with this finding and supports the concept that SLD amplifies acute peri-procedural vulnerability rather than driving long-term disease progression in this revascularized non-diabetic population. Although LVEF was included as a PSM covariate, its post-match SMD of 0.101 indicates marginal residual imbalance attributable to sparse and differentially available echocardiographic documentation. Importantly, the direction of this imbalance, with the SLD cohort having a slightly higher mean LVEF (55.8% vs. 54.4%), indicates that residual confounding from baseline systolic dysfunction would more likely attenuate than inflate the observed heart failure association, strengthening rather than undermining the biological plausibility of this finding.

The significant early cardiogenic shock excess (RR, 1.45; *p* < 0.001), attenuating to null beyond 30 days and across the full year, similarly supports a peri-procedural vulnerability mechanism. The cardiogenic shock endpoint was defined by ICD-10 code R57.0 alone, which may not capture all clinical presentations of hemodynamic compromise; this is a limitation in endpoint specificity but also suggests a conservative approach.

### Bleeding risk and antithrombotic implications

The significant association between coded SLD and one-year bleeding events (HR, 1.35; 95% CI, 1.15–1.59; *p* < 0.001) is the most clinically actionable finding of this study Fig. 2A. Unlike the hemodynamic outcomes, bleeding risk showed a consistent pattern across both early and late periods, suggesting that MASLD-associated hemostatic abnormalities, including platelet dysfunction, altered coagulation factor synthesis, enhanced fibrinolysis, and systemic inflammation, persist beyond the acute procedural window. This sustained hemorrhagic risk has direct implications for antithrombotic decision-making throughout the post-PCI year, not only in the immediate peri-procedural period.

Castiello et al. [26] emphasized in a comprehensive review that bleeding risk must be formally and individually assessed before and after PCI, and that prolonged DAPT in high-bleeding-risk patients may increase bleeding without parallel ischemic benefit. The sustained hemorrhagic risk profile of SLD patients supports individualized antithrombotic planning, including consideration of earlier DAPT de-escalation, radial access where feasible, and vigilant bleeding surveillance across the full post-procedural year. The hemorrhage composite used here does not permit BARC or TIMI classification, limiting interpretation of severity; these findings should be considered hypothesis-generating pending validation with standardized endpoints in patient-level datasets.

### Null findings for mortality, MI, 3P-MACE, cardiogenic shock, and cardiac arrest

The absence of statistically significant differences in all-cause mortality, MI, 3P-MACE, cardiogenic shock, and cardiac arrest over the full one-year period is not unexpected and requires careful interpretation in the context of the temporal analysis and the specific clinical setting of post-PCI care in a non-diabetic population.

For cardiogenic shock, the null overall finding (HR, 1.09; *p* = 0.463) despite a clinically meaningful early-period excess (RR, 1.45; *p* < 0.001) reflects temporal dilution: a concentrated early vulnerability that attenuates as the cohort stabilizes hemodynamically beyond 30 days. This pattern is biologically plausible as the peri-procedural period imposes peak physiological stress on a myocardium already potentially compromised by subclinical SLD-associated cardiomyopathy, and patients who survive this window are a selected, more resilient group. The attenuation of the cardiogenic shock signal beyond 30 days should therefore not be interpreted as the absence of risk, but rather as risk that may be front-loaded and time sensitive Fig. 1.

For all-cause mortality, the null overall result (HR, 0.93; *p* = 0.472) and the nominally lower late-period mortality in the SLD cohort (RR, 0.76; *p* = 0.040) most plausibly reflect a survivor enrichment effect: patients with SLD who experienced early cardiogenic shock or hemodynamic compromise and did not survive are no longer at risk in the late window, leaving a healthier survivor stratum that paradoxically appears to perform comparably or better than comparators. This phenomenon, well-recognized in observational post-ACS cohorts, can mask true underlying risk when analyzed across the full follow-up period without temporal stratification.

For MI, the null overall result (HR, 0.94; *p* = 0.533) is most plausibly explained by the success of revascularization in addressing the culprit lesion, which substantially reduces the substrate for recurrent spontaneous MI in the months following PCI. The early peri-procedural MI signal (RR, 1.07; *p* = 0.019) likely reflects type 4a periprocedural MI and index hospitalization coding rather than independent new ischemic events, and its attenuation beyond 30 days is consistent with effective revascularization rather than a true biological equivalence between groups. The null 3P-MACE result mirrors this pattern, as MI is a component of that composite.

For cardiac arrest, the null finding across all time windows is consistent with the overall mortality result and likely reflects the same survivor dynamics described above, compounded by the relatively low absolute event rate limiting statistical power to detect modest between-group differences.

Taken together, at least four competing explanations for the overall null findings merit equal consideration and transparency: temporal dilution of front-loaded risk, survivor enrichment in the late period, successful revascularization eliminating the culprit substrate for recurrent ischemia, and residual confounding from unavailable procedural variables — particularly PCI indication, lesion complexity, and completeness of revascularization — that are central determinants of post-PCI prognosis and cannot be recovered through propensity matching on administrative data alone.

### Strengths

Several methodological and design features strengthen the validity and clinical relevance of this analysis. First, the matched sample size of 7,434 patients per group, drawn from 67 healthcare organizations across the TriNetX US Collaborative Network, provides substantial statistical power and broad geographic and institutional representation, enhancing the generalizability of findings within the EHR-based study context. Second, the restriction to non-diabetic adults is a deliberate and important design choice: by excluding diabetes, which is one of the most powerful and well-established determinants of post-PCI prognosis. This study isolates the independent contribution of coded steatotic liver disease to outcomes, addressing a specific gap that prior studies conflated by including diabetic patients. Third, the propensity score matching framework was unusually comprehensive, incorporating not only standard cardiovascular comorbidities but also individual P2Y12 inhibitor agents (clopidogrel, ticagrelor, prasugrel), anticoagulants, SGLT2 inhibitors, GLP-1 receptor agonists, baseline ACS type, liver fibrosis, and left ventricular ejection fraction where documented, covariates that are frequently omitted in administrative database studies and that directly influence both ischemic and hemorrhagic outcomes after PCI. Fourth, the prespecified temporal stratification into early (day 0–30) and late (day 31–365) windows provides granular characterization of the post-PCI risk trajectory that a conventional single-window analysis would obscure, allowing distinction between acute peri-procedural vulnerability and sustained longer-term risk, a clinically meaningful distinction with direct implications for monitoring and management intensity. Fifth, the expanded bleeding composite, incorporating procedural hemorrhage, intracranial bleeding, and gastrointestinal hemorrhage codes beyond the narrow definitions used in prior administrative studies, captures a broader and more clinically relevant hemorrhagic risk profile, even if formal BARC or TIMI severity classification remains beyond the capabilities of the platform.

### Limitations

Several important limitations require consideration. First, administrative ICD-10 coding (K76.0, K75.81) does not capture the full contemporary MASLD definition, fibrosis stage, or disease severity [22]. Second, the comparator group likely includes individuals with undetected SLD, biasing results toward the null effect [22]. Third, key procedural determinants of PCI outcomes, PCI indication, lesion complexity, access site, stent type, DAPT duration, and antithrombotic intensity were unavailable; their absence means reported associations must be interpreted as conditional on available covariates only, and the term ‘independently associated’ is used with this caveat [26]. Fourth, although LVEF was included as a PSM covariate, echocardiographic data were available in only a subset of patients (14.3% of the SLD cohort and 13.2% of comparators after matching), and the post-match SMD of 0.101 marginally exceeded the prespecified balance threshold of 0.10. This residual imbalance reflects differential documentation frequency across cohorts rather than systematic physiological disparity. Notably, the SLD cohort had a slightly higher mean LVEF (55.8% vs. 54.4%), meaning the direction of imbalance would be expected to attenuate rather than inflate the observed heart failure and cardiogenic shock associations. Residual confounding from unmeasured echocardiographic parameters beyond LVEF, including diastolic dysfunction grade, left ventricular mass index, valvular disease, and right ventricular function, cannot be excluded. Fifth, although GLP-1 receptor agonist and SGLT2 inhibitor use was well-balanced across matched cohorts and included as propensity score covariates, subgroup analyses by these agents were not performed. This represents a meaningful gap, given that GLP-1 receptor agonists have been associated with improved left ventricular ejection fraction and reduced infarct size in patients with MI undergoing PCI, and SGLT2 inhibitors have demonstrated reductions in heart failure hospitalizations following acute MI even in non-diabetic populations [27]. Whether these therapies attenuate the hemodynamic vulnerability or hemorrhagic risk observed in our SLD cohort remains unknown and warrants dedicated prospective evaluation. Sixth, bleeding outcomes were not categorized using standardized severity scales such as the BARC criteria. Seventh, the high early MI and MACE rates reflect peri-procedural coding and the broad outcome window, not independent new events. Eighth, formal competing-risk analysis was not feasible given the aggregate-level TriNetX data structure. Ninth, findings from an academic healthcare network may not generalize to community settings.

## Conclusions

In this large propensity score–matched cohort of non-diabetic adults undergoing PCI, coded steatotic liver disease was associated with significantly higher one-year rates of incident heart failure (HR, 1.15) and bleeding events (HR, 1.35), without a significant difference in all-cause mortality. Time-stratified analysis reveals that hemodynamic vulnerability, particularly cardiogenic shock and heart failure, is concentrated in the early peri-procedural period (day 0–30), while hemorrhagic risk shows a more sustained pattern extending across the full post-PCI year. These findings characterize a clinically meaningful dual risk profile: acute peri-procedural hemodynamic fragility and persistent hemorrhagic vulnerability. They support heightened early post-procedural surveillance, individualized assessment of bleeding risk, and thoughtful antithrombotic management informed by current evidence-based frameworks [26] for patients with coded steatotic liver disease undergoing PCI. Prospective validation incorporating histological liver staging, echocardiographic characterization, and procedural-level data is necessary before these findings can be formally integrated into standardized risk stratification.

## Supplementary Information

Supplementary Material 1.

## Data Availability

The datasets analyzed during the current study were accessed through the TriNetX Research Network. Due to data use agreements and privacy policies, these data are not publicly accessible. However, access may be granted by TriNetX upon reasonable request and subject to institutional approval and applicable agreements.

## Abbreviations

AMI: Acute myocardial infarction
BARC: Bleeding academic research consortium
CI: Confidence interval
CPT: Current procedural terminology
DAPT: Dual antiplatelet therapy
HER: Electronic health record
GLP-1: Glucagon-like peptide-1
HIPAA: Health insurance portability and accountability act
HR: Hazard ratio
LVEF: Left ventricular ejection fraction
MASLD: Metabolic dysfunction-associated steatotic liver disease
MI: Myocardial infarction
NAFLD: Nonalcoholic fatty liver disease
NASH: Nonalcoholic steatohepatitis
RR: Risk ratio
SD: Standard deviation
TIMI: Thrombolysis in myocardial infarction
