# Supplementary material for: Effect of coded steatotic liver disease on outcomes of percutaneous coronary intervention in non-diabetic adults: insights from a multicenter real-world cohort
Source: BMC Cardiovasc Disord. 2026 May 25;26:643. doi: 10.1186/s12872-026-06017-y (PMC13417839; doi:10.1186/s12872-026-06017-y)

**Supplementary Table S1. ICD-10-CM Diagnosis Codes, CPT Procedure Codes, and Outcome Definitions**

Coded steatotic liver disease and PCI outcomes in non-diabetic adults — TriNetX US Collaborative Network (2014–2023)

| Code | Description | Role in Study |
| --- | --- | --- |
| Part A. CPT Procedure Codes — Percutaneous Coronary Intervention (Index Event) | | |
| 92920 | Percutaneous transluminal coronary angioplasty; single major coronary artery or branch | PCI — angioplasty, single vessel |
| 92921 | Percutaneous transluminal coronary angioplasty; each additional branch of a major coronary artery (add-on) | PCI — angioplasty, additional branch |
| 92928 | Percutaneous transcatheter placement of intracoronary stent(s), with coronary angioplasty when performed; single major coronary artery or branch | PCI — stent placement, single vessel |
| 92929 | Percutaneous transcatheter placement of intracoronary stent(s), with coronary angioplasty when performed; each additional branch of a major coronary artery (add-on) | PCI — stent placement, additional branch |
| 92933 | Percutaneous transluminal coronary atherectomy, with intracoronary stent, with coronary angioplasty when performed; single major coronary artery or branch | PCI — atherectomy with stent, single vessel |
| 92924 | Percutaneous transluminal coronary atherectomy, with coronary angioplasty when performed; single major coronary artery or branch | PCI — atherectomy with angioplasty |
| 92937 | Percutaneous transluminal revascularization of or through coronary artery bypass graft (internal mammary, free arterial, venous), any combination of intracoronary stent, atherectomy and angioplasty, including distal protection when performed; single vessel | PCI — bypass graft revascularization |
| 92941 | Percutaneous transluminal revascularization of acute total/subtotal occlusion during acute myocardial infarction, coronary artery or coronary artery bypass graft, any combination of intracoronary stent, atherectomy and angioplasty, including aspiration thrombectomy when performed, single vessel | PCI — primary PCI for acute MI |
| 92943 | Percutaneous transluminal revascularization of chronic total occlusion, coronary artery, coronary artery branch, or coronary artery bypass graft, any combination of intracoronary stent, atherectomy and angioplasty; single vessel | PCI — chronic total occlusion |
| 92972 | Percutaneous transluminal coronary lithotripsy (add-on) | PCI — coronary lithotripsy |
| Part B. Exposure Definition — ICD-10-CM Codes | | |
| Inclusion criteria (coded steatotic liver disease) | | |
| K76.0 | Fatty (change of) liver, not elsewhere classified | Exposure (SLD) — required |
| K75.81 | Nonalcoholic steatohepatitis (NASH) | Exposure (SLD) — required |
| Exclusion criteria (applied to both cohorts) | | |
| E08–E13 | Diabetes mellitus (all types) | Exclusion — both cohorts |
| K74.60 | Unspecified cirrhosis of liver | Exclusion — both cohorts |
| K70.* | Alcoholic liver disease | Exclusion — both cohorts |
| K72 | Hepatic failure, not elsewhere classified | Exclusion — both cohorts |
| Z94 / Z94.4 | Transplanted organ and tissue status / Liver transplant status | Exclusion — both cohorts |
| C00–C96, C7A, D00–D09 | Malignant neoplasms (all sites) and in situ neoplasms | Exclusion — both cohorts |
| Part C. Outcome ICD-10-CM Codes (Day 1–365 Post-PCI) | | |
| Primary outcome | | |
| — | Deceased (TriNetX vital status field) | All-cause mortality |
| Secondary outcomes | | |
| I21, I22 | Acute / subsequent myocardial infarction | MI — incident analysis |
| I50 | Heart failure | Heart failure — incident analysis |
| I21 + I63 + Deceased | MI, cerebral infarction, or death | 3-point MACE — incident analysis |
| R57.0 | Cardiogenic shock | Cardiogenic shock |
| I46 | Cardiac arrest | Cardiac arrest |
| Bleeding composite (13 codes — incident analysis) | | |
| K92.0 | Hematemesis | Bleeding composite — GI |
| K92.1 | Melena | Bleeding composite — GI |
| K92.2 | Gastrointestinal hemorrhage, unspecified | Bleeding composite — GI |
| I60 | Nontraumatic subarachnoid hemorrhage | Bleeding composite — intracranial |
| I61 | Nontraumatic intracerebral hemorrhage | Bleeding composite — intracranial |
| I62 | Other and unspecified nontraumatic intracranial hemorrhage | Bleeding composite — intracranial |
| R58 | Hemorrhage, not elsewhere classified | Bleeding composite — nonspecific |
| I97.41 | Intraoperative hemorrhage and hematoma of circulatory system organ complicating circulatory system procedure | Bleeding composite — procedural |
| I97.410 | Intraoperative hemorrhage and hematoma complicating cardiac catheterization | Bleeding composite — procedural |
| I97.411 | Intraoperative hemorrhage and hematoma complicating cardiac bypass | Bleeding composite — procedural |
| I97.418 | Intraoperative hemorrhage and hematoma complicating other circulatory system procedure | Bleeding composite — procedural |
| I97.42 | Intraoperative hemorrhage and hematoma of circulatory system organ complicating other procedure | Bleeding composite — procedural |
| I97.610 | Postprocedural hemorrhage of circulatory system organ following cardiac catheterization | Bleeding composite — procedural |
| Part D. Propensity Score Matching Covariates — ICD-10-CM Diagnostic Codes | | |
| Cardiovascular comorbidities and risk factors | | |
| I10 | Essential (primary) hypertension | PSM covariate |
| I25 | Chronic ischemic heart disease | PSM covariate |
| I50 | Heart failure | PSM covariate |
| I48 | Atrial fibrillation and flutter | PSM covariate |
| I63 | Cerebral infarction (history) | PSM covariate |
| E66 | Overweight and obesity | PSM covariate |
| E78.5 | Hyperlipidemia, unspecified | PSM covariate |
| E78.0 | Pure hypercholesterolemia | PSM covariate |
| N18 | Chronic kidney disease (all stages) | PSM covariate |
| K74 | Fibrosis and cirrhosis of liver (earlier-stage) | PSM covariate |
| I21.0 | STEMI of anterior wall | PSM covariate — ACS type |
| I21.1 | STEMI of inferior wall | PSM covariate — ACS type |
| I21.2 | STEMI of other sites | PSM covariate — ACS type |
| I21.3 | STEMI of unspecified site | PSM covariate — ACS type |
| I21.4 | Non-ST elevation (NSTEMI) myocardial infarction | PSM covariate — ACS type |
| I21.9 | Acute myocardial infarction, unspecified | PSM covariate — ACS type |

*Abbreviations: CPT = Current Procedural Terminology; ICD-10-CM = International Classification of Diseases, Tenth Revision, Clinical Modification; IVUS = intravascular ultrasound; OCT = optical coherence tomography; STEMI = ST-elevation myocardial infarction; NSTEMI = non-ST-elevation myocardial infarction; MACE = major adverse cardiovascular events; GI = gastrointestinal; PSM = propensity score matching; SLD = steatotic liver disease; ACS = acute coronary syndrome.*


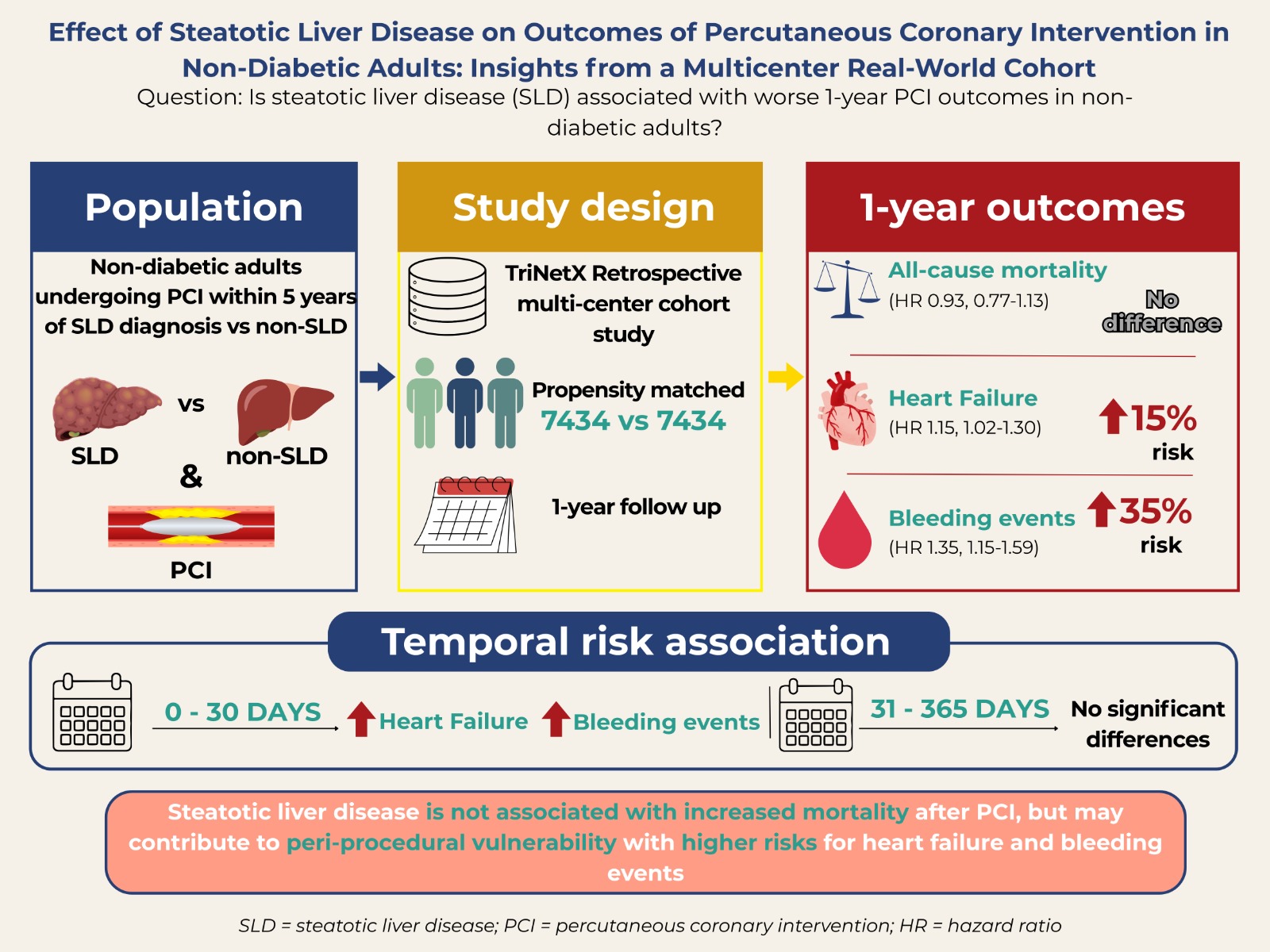

Supplement: Supplementary file 1 — Supplementary Material 1. [file 12872_2026_6017_MOESM1_ESM.docx]
